# Structural Features of 5′ Untranslated Region in Translational Control of Eukaryotes

**DOI:** 10.3390/ijms26051979

**Published:** 2025-02-25

**Authors:** Elizaveta Razumova, Aleksandr Makariuk, Olga Dontsova, Nikita Shepelev, Maria Rubtsova

**Affiliations:** 1 Chemistry Department, Lomonosov Moscow State University, Moscow 119234, Russia; elizaveta_razumova@list.ru (E.R.); olga.a.dontsova@gmail.com (O.D.); nikita.shepelev96@gmail.com (N.S.); 2 Department of Biology, Lomonosov Moscow State University, Moscow 119234, Russia; yaamakariuk@gmail.com; 3 A.N.Belozersky Institute of Physico-Chemical Biology, Lomonosov Moscow State University, Moscow 119234, Russia; 4 Shemyakin-Ovchinnikov Institute of Bioorganic Chemistry, Russian Academy of Sciences, Moscow 117437, Russia; 5 Skolkovo Institute of Science and Technology, Center for Molecular and Cellular Biology, Moscow 121205, Russia

**Keywords:** 5′ UTR, translation, TOP, uORF, RNA G-quadruplexes, riboswitches, IRES, m^6^A, stem-loops, pseudoknots, lncRNA

## Abstract

Gene expression is a complex process regulated at multiple levels in eukaryotic cells. Translation frequently represents a pivotal step in the control of gene expression. Among the stages of translation, initiation is particularly important, as it governs ribosome recruitment and the efficiency of protein synthesis. The 5′ untranslated region (5′ UTR) of mRNA plays a key role in this process, often exhibiting a complicated and structured landscape. Numerous eukaryotic mRNAs possess long 5′ UTRs that contain diverse regulatory elements, including RNA secondary structures, specific nucleotide motifs, and chemical modifications. These structural features can independently modulate translation through their intrinsic properties or by serving as platforms for trans-acting factors such as RNA-binding proteins. The dynamic nature of 5′ UTR elements allows cells to fine-tune translation in response to environmental and cellular signals. Understanding these mechanisms is not only fundamental to molecular biology but also holds significant biomedical potential. Insights into 5′ UTR-mediated regulation could drive advancements in synthetic biology and mRNA-based targeted therapies. This review outlines the current knowledge of the structural elements of the 5′ UTR, the interplay between them, and their combined functional impact on translation.

## 1. Introduction

Modern advances in molecular biology have unveiled the sophisticated nature of gene expression regulation within cells. Conventionally, the primary level of expression control in eukaryotes was presumed to be the transcriptional stage, which directed the majority of studies on transcriptional regulation. However, accumulating evidence indicates that the post-transcriptional stages frequently play a pivotal role in regulating gene expression. Among them, translation serves as the most significant post-transcriptional stage of expression regulation.

The regulation of eukaryotic translation predominantly occurs at the initiation stage. This process begins with the assembly of an initiation complex on the 5′ untranslated region of mRNA (5′ UTR). The 5′ UTR can harbor various complex RNA secondary structures, including hairpins, RNA quadruplexes, and pseudoknots, as well as the elements associated with non-canonical translation initiation mechanisms such as internal ribosome entry site (IRES). In addition, specific nucleotide sequences, including the terminal oligopyrimidine tract (TOP), upstream open reading frames (uORFs), and translation initiators of short 5′ UTRs (TISUs), play important roles. The regulatory complexity is further complicated by a variety of RNA modifications that also affect the efficiency of translation initiation.

The structural features of 5′ UTRs can act as independent regulators of translation due to their spatial configurations, or alternatively, to serve as a platform to attract trans-acting factors such as RNA-binding proteins. These structural elements possess a dynamic character that enables the cell to adapt to the translation processes in a flexible manner in response to external stimuli. Furthermore, the regulation at the translational level allows the cell to modulate gene expression more rapidly, as the transcription step is not required.

The present review summarizes current data on the structural elements of the 5′ untranslated region of mRNA, their influence on translation, and the interrelationships between them.

## 2. 5′ Untranslated Region in Translation Initiation

In most mature eukaryotic mRNAs, three functional regions can be identified: the 5′ untranslated region (5′ UTR) containing the 5′ cap, the coding region (CDS), and the polyadenylated 3′ untranslated region (3′ UTR). The sequences of eukaryotic 5′ UTRs have a high GC content relative to the average in mRNA, which may contribute to the formation of complex secondary structures [1]. The length of this region differs between organisms, with a median length ranging from 53 nucleotides in budding yeast to 218 nucleotides in humans [2]. However, it should be noted that the length of 5′ UTRs varies considerably even among transcripts from the same organism [2]. Notably, a positive correlation has been observed between the structural complexity of 5′ UTRs and gene significance [3]. It is also noteworthy that there are mRNAs with very short 5′ UTRs (12 nucleotides on average) that are translated without scanning with a special TISU regulatory element [4], as well as those completely lacking 5′ UTRs, such as mammalian mitochondrial mRNAs [5] and circular mRNAs. It should be clarified that each mRNA molecule is generally utilized in multiple rounds of translation, thereby determining the number of protein molecules produced. This phenomenon is referred to as translational heterogeneity, which can be either intergenic (mRNAs from different genes are translated differently) or intragenic (mRNAs from the same gene are translated differently within the same cell or in different tissues) [6]. Translational heterogeneity can be achieved by tuning the 5′UTR.

**Figure 1:**
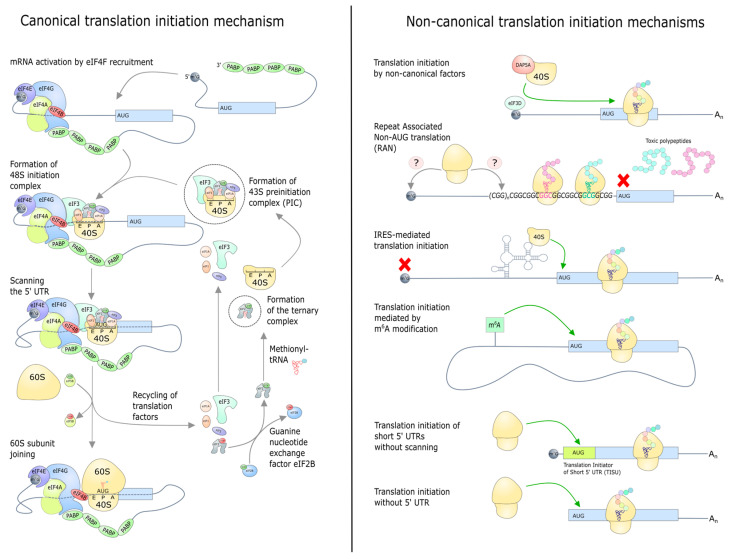
The canonical mechanism of translation initiation is illustrated on the left. This process includes the following stages: mRNA activation, the formation of the scanning 48S preinitiation complex, the recognition of the start codon, and the recruitment of the 60S subunit. On the right is a depiction of non-canonical translation initiation mechanisms. These include the use of non-canonical factors, various cap-independent mechanisms, and the translation of mRNA with very short 5′ UTRs.

Canonical translation initiation (Figure 1) begins with “mRNA activation”, the key step of which is the formation of a closed mRNA structure [7]. In a simplified model, during this process, the complex of initiation factors eIF4 binds the 5′ cap and polyadenylated 3′ end of the mRNA. The eIF4E protein interacts directly with the 5′ cap and the PABP protein binds to the poly-A tail. The integration of these elements into a unified complex is facilitated by eIF4G, thereby forming the ring structure of the mRNA. The eIF4A helicase, which is required for unwinding mRNA secondary structures, also binds to the complex, and its activity is regulated by the eIF4B and eIF4H proteins. Consequently, the eIF4E, eIF4G, and eIF4A proteins collectively form the eIF4F complex, which plays a central role in the mRNA activation.

In parallel with these processes, the 43S preinitiation complex is formed. The small 40S subunit of the ribosome, which is released after the completion of a translation cycle, binds to eIF3, eIF1, and eIF1A. Concurrently, a ternary complex comprising eIF2, GTP, and the initiator Met-tRNA_i_^Met^ is formed independently. These complexes, in combination with eIF5, form the interaction-ready 43S complex. Following this, the 43S complex binds to the activated mRNA by eIF3, assisted by eIF4F, forming the 48S complex. This complex then begins scanning the mRNA from the 5′ to 3′ end in search of the start codon. When the small subunit encounters the start codon in a favorable context, hydrolysis of GTP bound by eIF2 occurs, mediated by eIF5. Consequently, the large 60S subunit of the ribosome is attached to the small subunit by the GTPase eIF5B. This process is accompanied by the release of the initiation factors eIF1, eIF2, eIF3, and eIF5 and the hydrolysis of GTP bound to eIF5B, after which eIF1A and eIF5B also leave the complex. These processes result in the formation of a functional 80S ribosomal complex ready for translation elongation.

In addition to the well-established process of classical cap-dependent translation initiation, non-canonical mechanisms have been identified in eukaryotic cells that are activated when eIF4E-dependent translation is repressed, for example, under stress conditions (Figure 1). Structures such as internal ribosome entry sites (IRESs) and N6-methyladenosine (m^6^A) modification can recruit initiator factors and ribosomes independently of the 5′ cap. The cap-dependent translation can also be initiated by alternative initiator factors such as DAP5 and eIF3D [8]. A distinct mechanism is RAN (repeat-associated non-AUG), which initiates translation on GC-rich repeats [9]. This process occurs without an AUG start codon and leads to the accumulation of toxic proteins associated with neurodegenerative diseases. While the precise mechanism of RAN translation remains to be fully elucidated, the role of ribosome quality control proteins in mitigating the accumulation of its products has been identified, rendering them promising therapeutic targets [10,11].

## 3. Regulatory Sequences of 5′ Untranslated Region

### 3.1. Terminal Oligopyrimidine Tract (TOP)

The terminal oligopyrimidine tract (TOP) is a structure located immediately downstream of the m^7^G cap. In the classical TOP sequence, a cytidine residue is always present at position +1, followed by a continuous tract of 4-15 pyrimidines, followed by a GC-rich region [12]. Recent data show that the consensus TOP motif is shorter than previously thought and is 5′-**C**YYYYYY-3′ (where Y is C or U), and the sequence downstream is not important [13,14].

TOP is present in the mRNAs of all human ribosomal proteins and in several other genes encoding proteins directly related to translation (e.g., eIF3, eIF4A, eIF2, and PABP) [12]. The motif has also been identified in the mRNAs of proteins outside the translation apparatus (e.g., RACK1, HNRNPA1, nucleophosmin, NAP1L1, vimentin, TCTP, Tudor-SN, TATA Box-Binding Protein-Associated Factor 1D, and Galectin-1) [12,14,15], which may indicate their translation-associated function. It is noteworthy that numerous such transcripts are linked to oncogenesis [15,16,17,18,19,20,21,22]. In addition to the classical TOP, there are pyrimidine-rich translation elements (PRTEs) that undergo similar regulation to TOP [23]. The PRTE motif is defined by the presence of uridine at position 6 surrounded by pyrimidines (5′-YYYYY**U**YYY-3′), and its location is not immediately adjacent to the 5′ cap [23]. Currently, a transcript is categorized as a TOP based not only on its sequence but also on its relationship to translational regulation. Simplistic analysis of the terminal sequence reveals many more targets [24].

The TOP motif provides the selective regulation of gene translation depending on the energy and nutrient balance of the cell, mainly regulating components of protein biosynthesis [25]. In some cases, cooperative transcriptional and translational regulation is possible by switching between isoforms with different 5′ UTRs, which is important for tissue-specific expression and oncogenesis [13,26]. TOP regulation was initially hypothesized to be entirely dependent on the mTORC1 and PI3K pathways. However, subsequent studies have shown regulation independent of them [27,28,29], thus suggesting the involvement of a common trans-acting factor that integrates various cellular signals. Candidates for this role include S6K and rpS6, 4E-BP, La, LARP7, TIA-1/TIAR, CNBP/ZNF9, LARP1, miR-10a/miR-10b, and AUF1 [12,30]. However, the most compelling evidence has been obtained for the LARP1 protein. Interestingly, mRNAs that contain TOP are also susceptible to cytoplasmic recapping, a process that occurs in the cytoplasm subsequent to the partial degradation of the mRNA 5′ end [31]. Moreover, it has been observed that the position of recapping can be located downstream of the native site, thereby rendering the transcript immune to TOP regulation.

**Figure 2:**
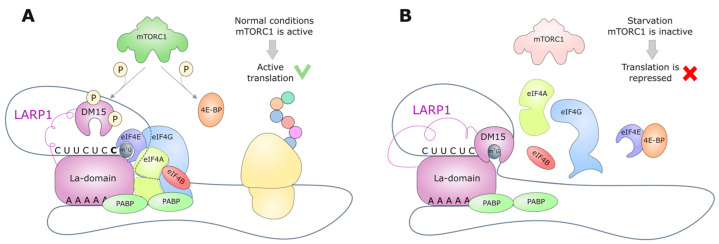
LARP1-mediated regulation of TOP mRNA translation. (**A**) The La-module of LARP1 binds the polyadenylated tail of mRNA and PABP independently of mTORC1. The DM15 domain of LARP1 is inactivated by phosphorylation, thus enabling the access of translation initiation factors to the 5′ end of TOP mRNA and its active translation. (**B**) The DM15 domain of LARP1, which is activated by dephosphorylation, blocks the assembly of the initiation complex through interaction with the TOP motif. This results in the TOP-mediated repression of mRNA translation under nutrient-deficient conditions.

The model of TOP motif regulation through LARP1, as proposed in 2020 (Figure 2) [30], has been corroborated by a number of studies. The La-module of LARP1 binds the polyadenylated tail of mRNA and PABP independently of mTORC1, and its DM15 domain, activated by dephosphorylation, blocks the assembly of the initiation complex through interaction with the TOP motif, thereby suppressing the translation of mRNA from TOP under nutrient deficiency [32]. In addition, eIF4A1 enhances the interaction between LARP1 and TOP, promoting translational repression [33]. It is also noteworthy that LARP1 possesses a number of regulatory functions unrelated to translation initiation. For instance, LARP1 regulates the length of polyadenylated tails through interaction with non-canonical poly(A) polymerases in response to amino acid starvation, with transcripts with longer poly(A) tails being preferentially translated [34,35]. It is important to note that LARP1 also affects the stability of TOP mRNA [36,37], forming complexes with TOP mRNA and the free 40S subunit of the ribosome, thus controlling the homeostasis of the translation apparatus [38,39,40]. Recent data on the yeast homolog of LARP1, Slr1p, indicate the universal nature of these regulatory mechanisms in eukaryotes [41].

### 3.2. Upstream Small Open Reading Frames (uORFs) and Start Codons

**Figure 3:**
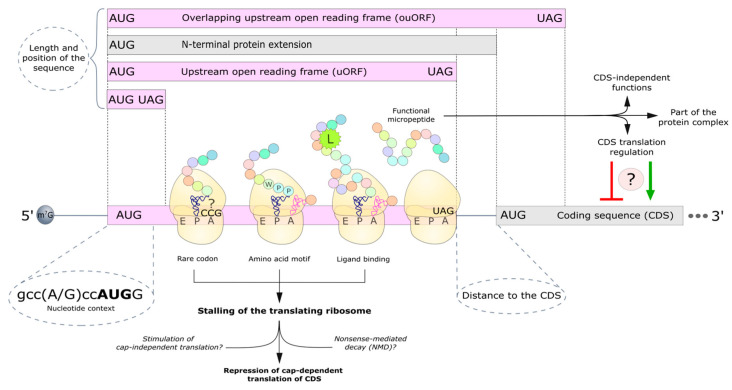
Regulation of translation by upstream ORF and start codons. The figure illustrates the characteristics of upstream ORFs (uORFs) that define their properties. Firstly, the location of the upstream start codon relative to the CDS and the resulting uORF length should be considered. The presence of an in-frame upstream start codon without an in-frame stop codon results in extended proteoform being translated. In all the other cases, uORFs are present. They can be located within 5′ UTR, or overlap the CDS (referred to as overlapping uORFs). The 5′ UTR can also contain start–stop regulatory elements. Secondly, the nucleotide context of the start codon, which determines the probability of translation initiation, and the distance to the CDS, which determines the probability of translation reinitiation at the CDS, also play a determining role. Moreover, uORFs can induce translation ribosome arrest in the case of a rare codon, a difficult-to-pass amino acid motif, or ligand binding by a nascent peptide. Ribosome arrest at the 5′ UTR can lead to mRNA degradation via the nonsense-mediated decay (NMD) pathway, stimulation of cap-independent translation, and repression of the CDS translation. uORFs may also encode a functional peptide that is part of the CDS product complex or has independent functions.

Other elements of 5′ UTRs that play a role in the regulation of translation in eukaryotes are upstream open reading frames (uORFs) (Figure 3). Typically, uORFs are short sequences (up to 300 nucleotides long) located between the start and stop codons. If the stop codon of the uORF is within the main coding region, such uORFs are classified as overlapping uORFs. The median length of uORFs is 60 nucleotides, though some are considerably shorter. Additionally, the 5′ UTR can contain regulatory elements consisting of only a start and a stop codon. These elements are distinguished from uORFs by the absence of the translation elongation step.

It is well established that the recognition of the start codon is dependent on the surrounding nucleotide sequence. The optimal consensus for translation initiation in eukaryotes is G^−6^C^−5^C^−4^(A/G)^−3^C^−2^C^−1^**A^+1^U^+2^G^+3^**G^+4^, with the purine base at position −3 playing a key role [42]. In a strong context, the AUG codon of uORF takes over part of the scanning ribosomes, thereby suppressing the translation of the main ORF (CDS). By contrast, the “leaky scanning” of the AUG by the 48S complex is possible in a weak context, which reduces the inhibitory effect of the uORF. Translation initiation can also occur at non-canonical start codons (CUG, GUG, UUG, etc.), but with significantly lower efficiency. Initiation factors are responsible for recognizing the strength of the nucleotide context of the start codon by the ribosome. eIF1 provides a more stringent selection, while eIF5 promotes initiation at codons with suboptimal contexts. Interestingly, the translation of these factors is regulated by uORFs [43]. Apart from uORFs, start codons located in the 5′ UTR and in the same reading frame as the main ORF can be used to produce alternative proteoforms of the protein, thereby altering the protein functionality [44,45,46].

Currently, the main tool for detecting uORFs is ribosomal profiling, which relies on the deep sequencing of small mRNA fragments protected by ribosomes from nucleases [47]. Depending on the compound used to stall the ribosome, the method can identify regions of the mRNA that contain elongating or initiating ribosomes. In addition, high-throughput full-genome screens based on the CRISPR/Cas9 system are used to select functional uORFs [48]. However, the studies conducted to date have primarily focused on the search for micropeptides and have not been used to study regulatory uORFs. Given the substantial accumulation of ribosomal profiling and peptidomics data, the current studies are directed toward the systematization and standardization of the annotation of all small open reading frames (ORFs), including uORFs, across diverse organisms [49,50,51,52]. This endeavor incorporates considerations of conservation, disease association, and other relevant factors.

Regulatory uORFs are frequently observed in the genes associated with cell differentiation, translation, oncogenesis, and stress response [43,53,54,55]. The conserved nature of many uORFs among mammals indicates their important role in the regulation of key processes whose disruption can lead to severe disease [56].

The length of the regulatory uORF affects its function: shorter uORFs minimize the loss of initiator factors to the ribosome that can be used in the reinitiation of translation [57,58]. The distance from the stop codon of the uORF to the start codon of the CDS is also critical: a longer distance increases the time to attract eIF2, which is required for methionyl-tRNA binding to the ribosome [57]. It is well established that under stress conditions, the phosphorylation of eIF2 leads to the formation of a stable complex of phosphorylated eIF2 with the GTP exchange factor eIF2B [59], which reduces the availability of the ternary complex (TC), thereby promoting the translation of the CDS of some mRNAs, for example, ATF4 [60,61]. Under conditions of abundant TC, the overlapping mammalian ATF4 uORF suppresses the translation of the CDS; in contrast, when TC is deficient, the scanning complex initiates translation at the CDS by skipping the overlapping uORF [62]. Recent evidence suggests that a hairpin located at the overlap of the uORF and the CDS of ATF4 may also be involved in controlling ATF4 translation [63]. The authors hypothesize that the hairpin may induce the formation of a queue of preinitiation complexes after the start codon of the CDS, thereby facilitating initiation at the start codon of the uORF. At the same time, the hairpin may also lead to the translation initiation of the CDS at the non-canonical CUG start codon located after the AUG [63].

Actually, the secondary structures of 5′ UTRs, including G-quadruplexes and hairpins, impede ribosome scanning, thereby mitigating leaky scanning and enhancing translation initiation at inhibitory uORFs [63,64,65]. Experiments by M. Kozak demonstrated that introducing a hairpin downstream of a weak start codon stimulates translation initiation [66]. Furthermore, the interaction of uORF with 5′ UTR elements has been observed to modify the 5′ UTR structure. For example, in the case of the arginine/lysine transporter cat-1, it has been shown that the translation of uORF presumably promotes the formation of an active IRES [67].

The inhibitory properties of uORF can be enhanced not only by increasing its length but also by altering its nucleotide sequence, including rare codons that slow down ribosomes [68]. For example, a recent study showed that the uORF inhibitory properties of the yeast transcription factor, known as Yap1, were found to be abolished by the expression of a rare codon tRNA [69]. Ribosome arrest can also be induced by encoded amino acid motifs. It is known that to overcome polyproline motifs, the ribosome requires the factor eIF5A with an important post-translational modification—the inclusion of the non-canonical amino acid hypusine, the precursor of which is the polyamine spermidine [70]. At the same time, polyamines compete with eIF5A for association with ribosomes, providing a negative feedback loop [71]. The 5′ UTR of the AZIN1 gene transcript, an activator of amine biosynthesis, contains an uORF with a conserved PPW motif [72]. When polyamines are in excess, eIF5A is unable to bind to the ribosome and assist it in overcoming the complex motif, resulting in the ribosome queue formed on the uORF and inhibiting translation of the CDS.

In addition, the translation of S-adenosylmethionine decarboxylase (AMD1) [73] mRNA is also modulated by polyamine levels via the uORF encoding the MAGDIS peptide [74,75]. AMD1 is involved in the regulation of spermidine biosynthesis. An increased level of polyamines has been demonstrated to result in the arrest of ribosomes in the proximity of the uORF terminal codon [76]. This phenomenon has been shown to inhibit the translation of the CDS [76]. It is hypothesized that polyamines may influence the interaction between the forming MAGDIS and the ribosome [77]. A similar mechanism has been observed for the translation of arginine-attenuator peptide (AAP) in *Neurospora crassa* and *Saccharomyces cerevisiae* [78]. The presence of arginine leads to a change in the conformation of this peptide in the ribosome tunnel and translation termination [79], which may contribute to the nonsense-mediated decay (NMD) of mRNA [80]. Other studies have also reported a possible relationship between NMD and the CDS in the 5′ UTR; nevertheless, the observed mRNA degradation may be due to other mechanisms [81,82]. It is interesting to note that in plants, uORFs frequently serve as metabolite sensors for a broad array of compounds, including sucrose, phosphocholine, ascorbate, thermospermine, galactinol, and others [83]. This suggests the intriguing possibility that numerous other sensor uORFs may be present in human cells.

Apart from their regulatory function, certain mRNAs encode functional micropeptides that modulate the activity of the CDS product or assume a role that is independent of it. For example, the CDS in the 5′ UTR of protein kinase C mRNA encodes a micropeptide uPEP2 (26 a.a.) possessing a pseudosubstrate motif [84]. Due to it, this micropeptide is capable of inhibiting not only the product of the CDS but also protein kinases belonging to other families. Another example is the micropeptide ASDURF, a subunit of the PAQosome chaperone complex [85], which is translated from the uORF of the *ASNSD1* gene and is required for medulloblastoma cell proliferation independently of the function of the ASNSD1 protein [86].

Numerous examples of the involvement of uORFs in translation leave no doubt about their importance as the regulatory elements of 5′ UTRs. Despite the large number of annotated uORFs, the detailed mechanisms of translation control involving them are poorly understood. The multistage and multifactorial nature of translation poses a significant challenge to the identification of all the possible regulatory pathways. Even the well-established model of the ATF4 translation regulation continues to be supplemented with new details. Despite the necessity for further research, the contemporary applications of data on uORFs already extend to biotechnology, especially plant engineering [87,88,89,90], and facilitate the explanation of the genetic basis of a number of human diseases [91].

## 4. RNA Structures Form Regulatory Elements in the 5′ Untranslated Region

### 4.1. RNA Secondary Structures: Stem-Loops and Pseudoknots

Intuitively, GC-rich 5′ UTRs should be capable of forming multiple stable structures such as hairpins (stem-loops) and pseudoknots. As discussed above, a hairpin downstream of the start codon can promote translation by increasing the time for AUG recognition by the ribosome, as in the case of *ATF4* [63]. Conversely, the presence of a stabilized hairpin may result in translation repression, as observed in the iron-responsive element (IRE) located in the 5′ UTR of ferritin [92,93]. This hairpin serves as the binding site for the iron-regulatory proteins IRP1/2, which stabilize it under iron-deficient conditions. It is noteworthy that hairpins have been observed to regulate non-canonical cap-dependent translation by binding eIF3 directly. The deletion of the binding site in the mRNA examples described, *JUN* and *BTG1,* results in the opposite effect on translation [94,95].

It is generally accepted that the prevalence of intricate secondary structures in 5′ UTRs impedes conventional cap-dependent translation by obstructing ribosomal scanning. However, a recent study employing mutagenesis revealed that the complex structure of the 5′ UTR of *SERPINA1* mRNA, contrary to the prevailing concept, promotes cap-dependent translation rather than inhibits it [96].

**Figure 4:**
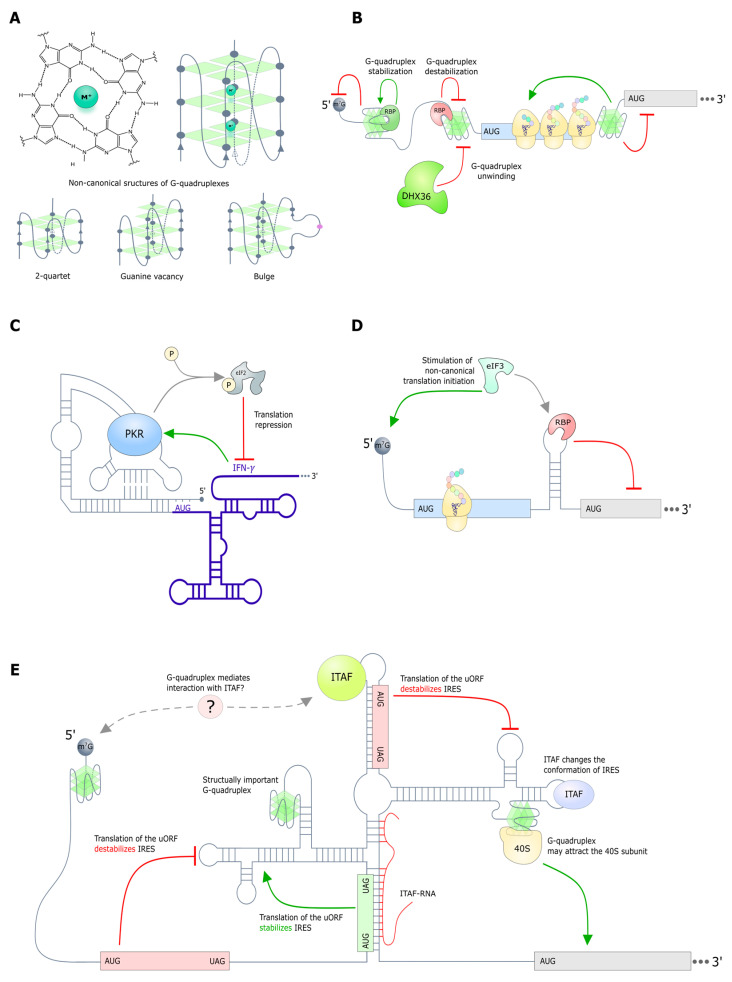
Regulation of translation by secondary structures. (**A**) The structure of canonical and non-canonical rG4. (**B**) The regulation of translation by rG4: Stable rG4 in the 5′ cap region or upstream of the start codon suppresses translation. Conversely, the presence of rG4 downstream of the start codon leads to a ribosome queue and promotes translation at the upstream codon. It is also worth noting that rG4 can be stabilized by RBP or unwound by helicases. (**C**) Translation can also be modulated by a pseudoknot: The translation of human IFN-γ mRNA containing a pseudoknot in the 5′ UTR is regulated by a protein kinase R (PKR) feedback loop. IFN-γ activates PKR via the pseudoknot, resulting in the increased phosphorylation of eIF2α and decreased IFN-γ translation. (**D**) A hairpin downstream of the start codon can promote translation by increasing the time for AUG recognition by the ribosome. Conversely, the presence of a stabilized hairpin may result in translation suppression, as observed in the iron-responsive element (IRE) located in the 5′ UTR of ferritin. This hairpin serves as the binding site for the iron-regulatory proteins IRP1/2, which stabilize it under iron-deficient conditions. Hairpins have also been observed to regulate non-canonical cap-dependent translation by binding eIF3 directly. The deletion of the binding site in the described mRNA examples, *JUN* and *BTG1,* have opposite effects on translation. (**E**) IRES regulation: The functionality of the IRES element depends on ITAF, rG4, and uORF. ITAFs modulate the conformation of IRES, typically resulting in its activation. RBP or lncRNA can act as ITAFs. rG4 can play a structural role in IRES, particularly in critical regions such as the 40S binding domain. It has been proposed that rG4 may facilitate the interaction of IRES with ITAF. The translation of an uORF upstream of IRES has been shown to suppress its activity. The translation of uORFs that are part of the IRES has been demonstrated to activate and repress it, promoting or inhibiting the formation of an active structure, respectively.

Translation can also be modulated by a pseudoknot, as evidenced by the observation that the translation of human IFN-γ mRNA containing a pseudoknot in the 5′ UTR is regulated through a protein kinase R (PKR) feedback loop [97]. IFN-γ activates PKR via the pseudoknot, resulting in the increased phosphorylation of eIF2α and decreased IFN-γ translation (Figure 4C). Despite the great potential of 5′ UTRs in the context of regulatory hairpin and pseudoknot formation [98], the number of detailed examples described is currently limited.

### 4.2. Riboswitches

Riboswitches are defined as structured RNA regions located in 5′ UTRs that regulate gene expression through the selective binding of different ligands [99]. In eukaryotic organisms, only a few examples of such structures have been described in chlorophytes and ascomycetes, all of which belong to the class of riboswitches sensitive to thiaminpyrophosphate, vitamin B1 [100]. These structures regulate splicing by altering the accessibility of splicing sites, depending on the amount of ligand, by changing the RNA structure. These structures were first described more than 10 years ago, and since then the discovery of alternative classes of riboswitches in eukaryotes has not been reported. Nevertheless, there is evidence for the existence of ligand-dependent regulatory structures in yeast 5′ UTRs. Specifically, studies have demonstrated that the 5′ UTRs of the *sam1* and *spe2* genes of *S. pombe* contain RNA structures that undergo conformational changes in response to ligand binding (SAM and spermidine, respectively), thereby regulating translation [101,102]. However, these findings require further validation.

The scarcity of extensive data on eukaryotic riboswitches may be partly due to the underdeveloped methodology for finding riboswitches in eukaryotic organisms. However, if this type of regulation is indeed less characteristic of eukaryotes and especially humans, a number of possibilities emerge. Firstly, a variety of riboswitches are widely distributed in bacterial genomes, where they regulate the expression of metabolite-associated genes [103]. They are currently being considered as potential targets for drug development to combat infections [104]. Secondly, the potential for the creation of various riboswitch-based regulated systems is introduced, which can be utilized for research purposes, the construction of viral constructs for transgene delivery, and synthetic biology [105,106,107,108].

### 4.3. G-Quadruplexes (rG4)

RNA G-quadruplexes (rG4s) are a class of extremely stable structures formed by guanine-rich sequences. rG4 consists of flat tetrads of four guanines linked together by Hoogsteen base pairs (Figure 4A). These tetrads form a negatively charged carbonyl oxygen center that coordinates positively charged cations, particularly K^+^, NH_4_^+^, and Na^+^. The cations K^+^ and NH_4_^+^ are large, and their location between the two tetrads stabilizes the quadruplex, in contrast to the relatively small Na^+^, which is embedded in the middle of one tetrad. Despite the fact that the existence of rG4 was demonstrated as early as 1991 in vitro [109], their presence and functionality in living cells remains a subject of debate, as the majority of studies have been conducted on synthetic oligonucleotides.

The classical sequence potentially forming rG4 has the following motif: G_X_-N_1-7_-G_X_-N_(1-7)_-G_X_N_(1-7)_-G_X_, where x is 3-6 nucleotides and N corresponds to any nucleotide (A, G, C, T, or U in the case of RNA). However, the current approaches also include the analysis of non-canonical rG4 in silico (Figure 4A). These are characterized by the presence of loops, bulges, the absence of some guanines, or the presence of only two tetrads [110,111,112]. Such non-canonical structures have been shown to dominate the transcriptome [110]. The distinguishing feature of rG4 from DNA quadruplexes is its increased stability due to the substitution of thymine for uracil and ribose in the sugar phosphate backbone. It is notable that these structural features also constrain the topology of rG4, restricting it to a predominantly parallel conformation, where all four strands are oriented in the same direction [113].

A wide range of methods are currently employed to detect G-quadruplex RNA, including modern high-throughput methods, such as rG4-seq, Keth-seq, SHALiPE, RT-stop, G4RP-seq, and others [114]. These methods provide the means to identify rG4 at the level of the entire transcriptome. The abundance of information has allowed the creation of databases dedicated to rG4 [112,115,116]. Experimental evidence has indicated that rG4 is enriched in the non-coding regions of mRNA, thus implying its significant functional role in the regulation of cellular processes [110], including transcription, mRNA maturation, and translational control [117]. The current data from high-throughput assays suggest that the rG4 found in vitro are predominantly unfolded in eukaryotic cells [118]. However, it cannot be excluded that most of them are dynamic structures, which mediate the active regulation of gene expression in response to changing conditions, as discussed in a recent review [119].

Detailed studies of rG4 are performed in vitro using synthetic oligonucleotides by physicochemical methods such as circular dichroism, UV spectroscopy, and NMR in the presence of stabilizing and destabilizing factors (metal ions, ligands, and aptamers) [114], and by chemical or enzymatic probing [114]. The development of a specific antibody BG4 was an important step in demonstrating the existence of rG4 in vivo [120]. However, the immunocytochemistry method has limitations because cell permeabilization can induce the formation of quadruplexes [113]. For this reason, organic chemistry efforts are currently focused on designing ligands that can be used to observe rG4 dynamics in a living cell [121,122,123,124,125,126,127,128]. Recent advances in method development also allow the use of NMR spectroscopy to study rG4 in vivo [129].

The effect of rG4 on translation is mainly studied using reporter constructs [130,131,132,133,134,135,136,137]. However, these approaches must be used with caution, as mutagenesis to destabilize rG4 can lead to changes in RNA structure, potentially impacting other regulatory regions in 5′ UTRs. This, in turn, can lead to either the inhibition or activation of translation. Intuitively, it is clear that stable rG4 located upstream of the open reading frame can interfere with ribosome movement and inhibit translation. This has been demonstrated using reporter constructs for multiple genes: *NRAS* [131], *TERF2* [132], *ATR* [133], *BCL2* [134], *ADAM10* [135], *CCND3* [136], *MMP16* [137], and *NRXN2* [130]. Moreover, using the 5′ UTR proto-oncogene *NRAS* as an example, it has been shown that this effect depends on the position and stability of rG4 [138]. The greatest effect is exerted by rG4, which is located closer to the 5′ cap and contains a large number of tetrads [138].

At the same time, in some cases, rG4 can promote translation. Most authors attribute the observed examples to cap-independent translation mediated by IRES. The rG4 are either directly part of the IRES structure or repress cap-dependent translation, thereby promoting IRES-mediated translation (discussed in detail in [139]). An example of rG4 located in the IRES structure is the quadruplex of the 5′ UTR of the *VEGF* gene [140]. Using footprinting, it was shown that rG4 located in the putative IRES of *VEGF* directly recruits the 40S subunit of the ribosome, thereby promoting cap-independent translation.

In addition, it has been reported that rG4 can stimulate translation independently of an IRES. For instance, one study suggests that the disruption of rG4, which is located near the 5′ cap of the cellular inhibitor of apoptosis protein cIAP1, significantly reduces translation levels [141]. Studies focusing on TGF-β2 gene have found that two quadruplexes in the 5′ UTR cooperatively enhance translation by an unknown mechanism [142,143]. Further studies are needed to understand the mechanisms of these effects, especially in the context of their association (or lack thereof) with IRES.

It is worth noting that rG4 can cooperate with other structures to affect canonical and cap-independent translation in different ways. For example, rG4, located near the cap in the 5′ UTR of the BAG-1 gene, suppresses cap-dependent translation by creating a structural obstacle for the ribosome. However, the rG4 simultaneously promotes IRES-mediated translation because its absence causes a change in the structure of the IRES located 300 nucleotides downstream [144].

The regulation of translation by rG4 in 5′ UTRs has been shown for various organisms: rG4 affects gene expression in malaria plasmodium, humans, and plants [65,145,146]. In addition, rG4 is commonly found in the 5′ UTRs of certain groups of genes, indicating a likely co-regulation of their expression. For example, rG4 has been shown to be enriched in the 5′ UTR mRNAs of ribosomal proteins in the GC-rich region following TOP, and their translation is regulated by specific helicases [147].

A number of helicases, such as DHX36, DHX9, DDX21, DDX2 (eIF4A), MOV10/L, and nucleic acid binding proteins, CNBP and hnRNP H/F, are known to bind and unfold rG4 [116,148,149,150]. For example, the DHX36 helicase is enriched in mRNA regions with rG4 and promotes quadruplex unfolding and translation. Under normal conditions, the expression level of DHX36 is low, but under certain conditions, such as the proliferation of skeletal muscle stem cells, it is upregulated, activating the translation of a group of genes that have rG4 in the 5′ UTR [151]. Later, the same authors showed that in the *ANP32E* gene, rG4 unwinding is promoted by the long non-coding RNA Lockd, which stabilizes the interaction between DHX36 and eIF3B [152]. High-throughput methods have confirmed the presence of rG4 downstream of multiple uORFs, where it promotes translation of these uORFs when DHX9 and DHX36 helicases are deficient [65]. Using fluorescent probes in vivo, rG4 has been shown to regulate ADAR translation in a DHX36-dependent manner [153]. Ribosome footprinting revealed eIF4A-mediated translational control of the rG4-containing transcripts of oncogenes [154].

rG4 in 5′ UTRs represent promising targets for therapy. Mutations that destabilize rG4 are associated with a number of diseases [155,156,157,158,159,160]. The development of targeted drugs that stabilize or destabilize the functional quadruplex, such as conjugates with aptamers [161] or small molecules [162], may allow the regulation of target gene expression. The fundamental possibility of this has been shown for the *KRAS* [163] and *NRAS* proto-oncogenes [164]. In addition, the role of rG4 in the stress response has been discussed in the literature [165]. In particular, a recent study reported that rG4 in the 5′ UTR of the *FEN1* gene may act as a sensor of reactive oxygen species by regulating the expression of this gene [166].

### 4.4. IRES

Another example of a structure that regulates translation is the internal ribosome entry site (IRES). These structures were originally found in viruses. They promote ribosome recruitment and the subsequent translation in a cap- and 5′ UTR-independent manner. Viral IRESs have been the subject of significant research, and their structures in complex with the ribosome have been thoroughly characterized. They are actively used in biotechnology to create polycistronic mRNAs for expression in eukaryotic cells [167].

However, the existence of “genuine” cellular IRES is still a matter of debate despite many publications and even the creation of cellular IRES databases [168]. The fact is that most of the data that are currently available have been obtained using methods that can lead to a large number of artifacts and, as a consequence, to false positive results. For example, when using DNA-based bicistronic reporters, the translation product of an alternative mRNA transcribed from a putative promoter hidden within the putative IRES is taken as the signal from the IRES-mediated translation product. Recently, a group of researchers published a detailed guide on how to use dual reporter systems and what controls are necessary to avoid such errors [169]. In addition, there is some terminological confusion between IRES and CITE (cap-independent translation enhancer) [170]. It is important to note that while many studies equate cap-independent translation with IRES-mediated translation, this is not correct. The difference is that the IRES directly provides ribosome assembly and promotes scanless translation, whereas CITE requires the 5′ UTR [170]. Therefore, even experiments that confirm the presence of cap-independent translation of a transcript do not prove the presence of IRES.

To date, several principal mechanisms of IRES regulation have been identified (Figure 4E). It is hypothesized that IRES can be regulated by ITAF (IRES trans-acting factors) and the aforementioned rG4 and uORFs. ITAFs are proteins or regulatory RNAs that change the conformation of the IRES in response to conditions, generally serving an activating function [171]. ITAFs are suggested to play an important role in the cell’s coordinated response to stress [171]. rG4 may be an important structural element of IRES that determines its activity, in particular by directly binding the ribosome, as has been shown for *VEGF* [139,140]. In addition, rG4 at the 5′ end of the *BAG1* gene has been reported to promote cap-independent translation, although it is not part of IRES. The authors posit that this rG4 may be crucial for stabilizing the IRES structure by recruiting ITAF [144]. uORFs may have the opposite effect on IRES-mediated cap-independent translation. As previously discussed, the translation of an uORF, as part of the IRES structure in cat-1 mRNA, changes the secondary structure to form an active IRES [172]. In contrast, the translation of uORFs within the IRES structure of *VEGF* suppresses its activity, as does the upstream IRES uORF in the *FGF9* gene [173,174]. Previously, it was assumed that the IRESs could be regulated by TIE (translation inhibition element) and attract specialized ribosomes in *HOXA9* [2,175]. However, a recent publication disputes the presence of IRES in *HOXA9* [176].

Current evidence suggests that IRES can integrate signals from various cis- and trans-acting factors, thereby mediating complex translational regulation. However, due to a number of methodological issues, the findings on cellular IRESs should be interpreted with caution. Nevertheless, the abundant evidence of the importance of genome regions containing putative IRESs highlights the need for a detailed study of these structures. It cannot be excluded that these regions may in fact represent CITEs or other regulators of translation or transcription, a possibility which does not diminish their scientific value.

## 5. Modifications of mRNA

**Figure 5:**
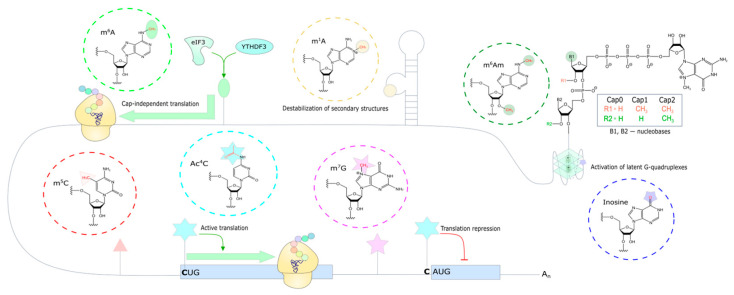
Diversity of modifications in the 5′ UTR of mRNA and their functions. The 5′ UTR and eukaryotic cap variants exhibit a variety of modifications. m^6^Am is found in the nucleotide following the cap. Inosine is a potential source of latent rG4, while m^1^A has been linked to the destabilization of secondary structures. m^6^A has been shown to contribute to non-canonical translation initiation. m^5^C and internal m^7^G have been detected in the 5′ UTR of eukaryotes, though their specific functions remain to be elucidated. Ac^4^C is believed to promote translation at the CUG start codon and repress translation at position −1 of the AUG.

Previously, multiple nucleotide modifications were thought to be characteristic of non-coding transcripts. However, recent data indicate a wide variety of mRNA modifications (Figure 5) [177]. In addition to the well-established methylation of the N7-position of guanosine at the 5′ end of mRNA, the development of methods has enabled the identification of modifications such as m^6^A, m^6^Am, m^1^A, ac^4^C, pseudouridine, m^5^C, inosine, and internal m^7^G in all the regions of mRNA. They have different functions depending on location and affect mRNA processing, stability, localization, and translation. These modifications are dynamically regulated by three classes of enzymes: writers, readers, and erasers [177].

One of the most significant structural elements of mRNA is the 5′ cap. This is a co-transcriptional modification of mRNA, during which **m^7^G** is added to the 5′ end, attached via a 5′-5′-triphosphate bond, forming “cap 0”. In higher eukaryotes, the nucleotide following the cap is methylated at the 2′-O position to form “cap 1”. This modification is crucial for distinguishing between host and foreign RNA [178]. In addition, a subset of vertebrate mRNAs has either m^6^A or 2′-O-dimethyladenosine (m^6^Am) as the first nucleotide. In higher eukaryotes, the second nucleotide also undergoes ribose methylation, forming “cap 2”. It is noteworthy that alternative complex caps derived from metabolites such as NAD have been observed, but their function remains to be fully elucidated [179]. Accumulated evidence suggests a variety of mechanisms of capping, in particular, cytoplasmic re-capping [178]. In addition to the recruitment of initiator factors and the protection of mRNA from degradation, the cap and modifications to the 5′ end of mRNA may serve as a platform for interaction with a number of RNA-binding proteins involved in translation regulation. It has been determined that there are internal m^7^G sites that are enriched in the 5′ UTR near the translation initiation site, which are conserved among mammals [180,181].

N6,2′-O-dimethyladenosine (**m^6^Am**) is a modified nucleotide following the cap in many mammalian mRNAs. Research findings concerning the effect of this modification on translation are contradictory. The available evidence suggests that the effect may be tissue and cell type-specific, and may also depend on the methylation of the nucleotide following it [182]. It is also noteworthy that a number of methods for mapping m^6^A and m^6^Am fail to distinguish between these modifications. For instance, it has been demonstrated that a number of m^6^Am sites following the cap have been erroneously annotated as internal due to alternative transcription start sites [183].

The most extensively studied mRNA modification is **m^6^A**, which is uniformly distributed throughout its length and is particularly enriched around the stop codon [177]. This modification plays a critical role in the regulation of mRNA splicing, stability, localization, and translation [184]. It has been demonstrated to promote cap-independent translation. Specifically, m^6^A can bind directly to eIF3 [185,186], engaging the 43S initiator complex, or mediate translation through reader proteins such as YTHDF3 [187]. The mechanism of translation initiation via m^6^A has been proposed as a model for the translation of circular RNAs [188]. It is noteworthy that the dynamic regulation of translation mediated by m^6^A contributes to the cell’s complex and adaptive response to stress [189].

Another mRNA modification, N1-methyladenosine (**m^1^A**), is an isomer of m^6^A in which the nitrogen atom at position N1 instead of N6 is methylated. m^1^A is able to alter base pairing in RNA, turning the Watson–Crick pair into a Hoogsteen pair [190], potentially disrupting RNA secondary structure. Data show that m^1^A is enriched in 5′ UTR cytoplasmic mRNAs, mainly at the translation initiation site [191,192]. Additionally, reports indicate that m^1^A nucleotides are enriched near alternative translation initiation sites and are found at positions +1 and +2 relative to the cap [193,194]. Moreover, m^1^A in 5′ UTRs has been shown to be positively correlated with translation efficiency [179,182]. It is acknowledged that m^1^A in 5′ UTRs is dynamically regulated under stress conditions, including oxidative stress and starvation, resulting in a significant increase in its content [192].

The **m^5^C** modification is enriched at the 5′ UTR and near the CDS start [195,196]. While m^5^C has been demonstrated to suppress translation, the observed effects are more likely to be associated with sites located in the CDS rather than in the 5′ UTR [195,196].

The conversion of adenosine to **inosine** is a rare occurrence in 5′ UTRs, being more prevalent in 3′ UTRs [197]. However, it is noteworthy that the replacement of adenosine by inosine results in the activation of “latent” rG4 [198]. rG4 are observed in both 3′ and 5′ UTRs, thereby highlighting the potential significance of inosine in the regulation of 5′ UTRs.

The presence of the **ac^4^C** modification in human mRNA is currently a matter of debate in the scientific community. In 2018, a group of researchers published data suggesting that ac^4^C is distributed throughout human mRNA, with an enrichment observed in the 5′ UTR near the translation initiation site [199]. Subsequent studies by the same authors revealed that the impact of ac^4^C on translation depends on the context, promoting translation initiation at non-canonical start codons (CUG) in 5′ UTRs and inhibiting translation in Kozak (AUG) contexts [200]. However, an opposing study in 2020 claims that ac^4^C is not present at all in human and yeast mRNA [201]. This discrepancy underscores a need for further investigation into the role of ac^4^C modification in eukaryotic RNA.

The current state of knowledge regarding the effects of different nucleotide modifications in 5′ UTRs on translation is limited due to a number of instrumental problems [202]. Firstly, the mapping of different modifications across the entire transcriptome is challenging for a number of reasons, including methodological artifacts and annotation errors. Secondly, even reliable data does not provide a comprehensive understanding of dynamic regulation. The current approaches utilize methyltransferase knockouts, which only allow changes in modification status to be seen across the entire transcriptome. Additionally, the study of site-specific regulation is hindered by the fact that the same enzyme typically introduces modifications at different positions and a change in its expression can lead to a global rearrangement of processes in the cell. However, the enrichment of modifications near functionally important 5′ UTR regions, such as the translation initiation site, indicates that they may play an important role in the regulation of gene expression and this aspect requires further study.

## 6. Trans-Acting Factors

As discussed in previous sections, 5′ UTR-mediated translation regulation by trans-acting factors involves various elements, such as the coupling of LARP1 and TOP, ITAFs, and IRES. In addition to these examples, it is important to consider cases where the interaction is sequence-determined. This includes a variety of RBPs that recognize specific sequences within the 5′ UTR, as well as complementary long non-coding RNAs (lncRNAs) and microRNAs (miRNAs).

For example, HuD, an RNA-binding protein, has been shown to be associated with a 22-nucleotide segment of the 5′ UTR of preproinsulin [203]. The overexpression of HuD suppresses the translation of the insulin precursor, while glucose treatment causes the dissociation of HuD from *Ins2* mRNA and increases insulin production, respectively. It has been hypothesized that microRNA-196b can enhance insulin translation by binding to the 5′ UTR and displacing the HuD inhibitor [204]. Additionally, studies have demonstrated that the splicing factor SRSF3 not only regulates splicing but also suppresses the translation of its target PDCD4 by directly binding to the 5′ UTR [205]. The list of RNPs regulating translation continues to grow, and in silico evidence suggests that there is a wide range of multifunctional RBPs that bind 5′ UTRs [206,207]. However, it should be noted that such proteins often bind both ends of the UTR, and it is not yet known which interaction determines the regulation.

The most studied trans-acting long non-coding RNA (lncRNA) is the antisense RNA that controls the translation of Uchl1 [208]. This RNA has a complementary region to *Uchl1* mRNA and an “effector domain” that enhances translation. The non-complementary sequence of the RNA contains SINEs (short interspersed repetitive elements), specifically SINEB2 of subclass B3. This work initiated the study of a class of long non-coding RNAs (lncRNAs) called SINEUP: lncRNA translation enhancers containing SINEs. A number of other examples of antisense RNAs that regulate translation in this manner were later found, e.g., R12A-AS1 [209], Uxt [208], and AS-RBM15 [210]. However, the precise mechanism by which antisense RNAs enhance translation remains to be elucidated. SINEUPs have been observed to colocalize with their mRNA, facilitated by RNA-binding proteins PTBP1 and HNRNPK [211]. Additionally, studies have shown that m^6^A modification is necessary for optimal SINEUP function [212]. A recent study reported that SINEs can bind ribosomal RNA [213], and a recently published preprint suggests that the effector domain functions as an IRES [214]. Given the versatility of long non-coding RNA (lncRNA) with an antisense and effector domain structure, the potential of SINEUP in therapeutic applications to enhance gene expression is a promising avenue for further exploration [215,216,217].

## 7. Conclusions

The efficiency of translation is determined by a set of mRNA structural elements located in 5′ UTRs, which exert different, often opposite effects on the process of protein synthesis. These elements interact in a complex manner, allowing the cell to dynamically and precisely regulate translation in response to environmental changes, ensuring a high degree of adaptability. The accumulated data emphasize the need for a systems approach, including the integration of omics studies, to unravel the molecular mechanisms of translation regulation of individual genes. This comprehensive approach will facilitate a more profound understanding of how the structural characteristics of mRNAs influence the expression programs of diverse genes.

Beyond its fundamental importance, the study of the regulatory mechanisms of translation offers considerable potential for practical applications. This knowledge can be used to create new tools in synthetic biology, develop targeted therapy, and improve mRNA vaccine technology. The controlled translation of target mRNAs, predicated on an understanding of their structural features, promises to substantially expand the scope of their applications in biomedicine and biotechnology.

## Abbreviations

The following abbreviations are used in this manuscript:

UTR	untranslated region
IRES	internal ribosome entry site
TOP	terminal oligopyrimidine tract
uORF	upstream open reading frames
RAN	repeat-associated non-AUG (translation)
TISU	translation initiators of short 5′ UTRs
PRTE	pyrimidine-rich translation elements
rG4	RNA G-quadruplexes
CDS	Coding sequences
CITE	cap-independent translation enhancer
ITAF	IRES trans-acting factors
